# Predictors of non-adherence to antiretroviral therapy among HIV patients in secondary health care facilities in Kano State- Nigeria: a case-control study

**DOI:** 10.11604/pamj.supp.2019.32.1.13746

**Published:** 2019-01-21

**Authors:** Sudawa Aminu Usman, Adamu Shehu, Olufemi Ajumobi, Saheed Gidado, Ibrahim Dalhatu, Muhammad Balogun, Mohammed Riyad, Ibrahim Saude, Peter Adewuyi, Peter Nsubuga

**Affiliations:** 1 Nigeria Field Epidemiology and Laboratory Training Program, Nigeria; 2 Ahmadu Bello University, Zaria, Nigeria; 3 State House Medical Center, Abuja, Nigeria; 4 Aminu Kano Teaching Hospital, Kano, Nigeria; 5 Global Public Health Solutions

**Keywords:** Non-adherence, predictors, antiretroviral

## Abstract

**Introduction:**

Treatment success requires both a sustainable supply of Antiretroviral Therapy (ART) to clinics and lifelong adherence to treatment by patients. Poor adherence to medication may lead to treatment failure as a result of developing HIV resistance strains. Based on WHO 2014 guideline, over 26 million people will be additionally enrolled globally. Optimal treatment requires identification of patients with suboptimal adherence for targeted intervention. The aim of the study was to determine the predictors of non-adherence to ART.

**Methods:**

An unmatched 1:2 case-control study with 68 cases using simple random sampling. A case was defined as an ART patient who failed to achieve increase in CD4 count of 100cell/mm^3^ in one year. Controls are those with adequate immunological response. Questionnaires were administered for socio-demographic and adherence-related information. Bivariate and multivariable logistic regression was done using Epi Info at 95% Confidence Interval (CI) and precision of 5%.

**Results:**

A total of 204 patients were enrolled, mean age of cases was 36 (Standard Deviation Sd=8.8), n=68 and controls 37 (Sd=9.8), n=136. Of enrollees, 69.6% (142) were females. Mean duration on ART was 4.9 years for cases and 4.5 years for controls (standard deviation = ± 2.2 years). The commonest reason for missing drugs was forgetfulness (58% cases). On multivariate analysis, having formal education (Adjusted Odds Ratio [aOR] = 3.03, 95% CI = 1.5-6.0) and income above minimum wage, (aOR = 2.38, 95% CI = 1.06-4.76) were independently associated with non-adherence. The commonest reason for missing drugs was forgetfulness (58% cases).

**Conclusion:**

In conclusion, educated people and those with higher income were more likely to be non-adherent and should be the focus of adherence counseling. Some form of reminders like use of telephone should be adopted to address forgetfulness.

## Introduction

Almost 75 million people have been infected with HIV since the beginning of the pandemic and about 36 million people had died by 2014. Globally, 35.3 million people were living with HIV at the end of 2012. The burden varies between countries and regions with sub-Saharan Africa being most affected where nearly 1 in every 20 adults is infected accounting for 71% of the global burden [1]. The use of Highly Active Antiretroviral Therapy (HAART) has transformed HIV from a rapidly fatal disease to a chronic medical condition. Adherence, on the other hand refers to the persistence and tenacity that patient needs to achieve in sticking to a therapeutic regimen [2]. In Nigeria HAART was introduced in 2002 and today ≥ 500,000 people are using HAART out of ≥ 1,500,000 eligible clients [3]. In Kano, the use of HAART was commenced in2005 and by the end of 2015 ≥ 27,000 patients were receiving Antiretroviral Therapy (ART). The new 2014 guidelines from the World Health Organization (WHO) recommend that people living with HIV should start ART much earlier and in some instances immediately upon diagnosis. Consequently, some 26 million people worldwide will now be eligible for ART, an additional 9.2 million from the previous 2010 guidelines [4]. Previous studies suggest that anti-retroviral drug resistance evolves largely as a result of poor adherence to drugs by patients and was attributed to extensive use of ART in the developed nations [5]. However ART scale up in resource-poor settings could accelerate the emergence of HIV drug resistance due to insufficient viral load monitoring, inconsistent drug supply and possible unregulated use of antiretroviral (ARV) drugs which could potentially reverse the gains recorded from the use of ART. Where non-adherence is wide spread, transmitted drug resistance (TDR) or primary resistance develop which increases the risk of virologic failure to first line ARTs [6]. In the context of limited treatment options, secondary resistance and TDR are important hurdles in the control of HIV pandemic [7]. Various reports have documented an increase in the development of ARV drug resistance in many countries and regions of the world [8].

Adherence plays a dominant role in determining treatment success among Persons Living With HIV (PLWH) and is influenced by factors separate from timely initiation of ART [9]. These factors differ from resource-rich to resource-poor settings because of adverse psychosocial factors more prevalent within specific demographic groups compared to the general population [10]. In the United States, women are more likely to fail treatment than men on the same medication. Determinants of poor adherence include non-white ethnicity, low education, income less than $10,000 per year, incarceration, being a single parent, alcohol abuse and psychiatric morbidity [11]. A study from Kwazulu-Natal South Africa in 2010 indicated that factors that favor adherence include urban residence, the absence of discrimination experience, better adherence information, not using herbal medicine and higher social support [12]. In Benin City south-eastern Nigeria it was reported in 2008 that poverty, medication adverse effects, confidentiality, occupational factors, stigma and discrimination were determinants of the level of adherence [13]. A similar study on determinants of adherence from Keffi Nasarawa State, north-central Nigeria in 2013 found that adherence was positively associated with disclosure of HIV status and living with family, patient’s educational level, marital status and occupation. Reasons for non-adherence were forgetfulness, side effects, living far away from the medical center and inability to afford the cost of transportation to the medical center [14]. Iliyasu *et al.*, from Kano showed that the educated patients were more likely to be compliant whereas age and sex variables had no significant influence on compliance. Reasons for non-adherence were non-availability of drugs, forgetfulness and lack of funds [15]. The aim of this study was to identify the determinants of ART non-adherence in Kano State to guide the State Ministry of Health in the implementation of appropriate strategies to address the problem.

## Methods

### Study area

The study was conducted at the Infectious Diseases Hospital (IDH) and Murtala Muhammad Specialist Hospital (MMSH) in Kano State Nigeria between April and May 2015. It is located in the northern part of the country, a cosmopolitan and commercial centre with the largest population estimated at 11,645,202 based on 2006 national census. It occupies 20,131 square kilometers and majority of the inhabitants are farmers and traders. Over 90% of the residents are Muslims and Hausa by tribe. IDH and MMSH have been the pioneer HIV treatment sites in Kano since 2005. The prevalence of HIV in Kano State was 2.2% based on 2012 national sentinel survey. There are 63 treatment centers offering HIV comprehensive care as of May 2015 with 27,000 clients are receiving ARV care in the State. At the time of the study, 15,232 were enrolled into HIV care in IDH Kano out of which 5,255 clients (2,234 males, 3,021 females) had commenced ART as from September 2014. The MMSH had a total of 4,093 clients (1,399 males, 2,694 females including 247 pediatrics) who were initiated on ART as from September 2014. The two hospitals accounted for 33% of HIV patients receiving ART in Kano State.

**Study design and population:** this was an unmatched case control study. The ratio of cases to controls is 1:2.

**Definitions:** a case was defined as anybody with CD4+ cell count below 50% of peak level after commencing treatment for at least 1 year or who fails to achieve increase in CD4 count of 100cell/mm3 above the base-line or a total CD4 cell count of < 200cells/mm^3^ after 1 year on ART. Controls were defined as clients with an adequate immunological response as characterized by CD4+ level (achieving a CD4 cell count of at least 100cells/mm^3^ or a total of > 200cell/mm^3^ in a year) or a decline in CD4 cells count to not less than 50% of peak level after at least 1 year on ART at the time of the study.

Clients were categorized as having good adherence if missing a maximum of three doses in 1 month for those on once a day regimen and four doses for those on twice a day regimen [16]. Inability to achieve this level of adherence was referred to as non-adherence. In this study, we considered that clients with poor immunological response (cases) had poor adherence whereas those with good immunological response (control) had good adherence. Drug holiday referred to missing the ARV for a longer period (> 1month). Minimum wage referred to an income of less than N18,000 ($90) in a month. Formal education refers to having at least a primary education.

**Inclusion criteria:** we recruited any client who was commenced on ART not later than April 2014 and fit into the definitions stated above for cases and controls.

**Exclusion criteria:** pregnant women and clients with autoimmune diseases, e.g., Systemic Lupus Erythromatosus, Dermatomyositis, Rheumatoid Arthritis, co-morbidities, minors and non-consenting clients were excluded. These clients are likely to have additional impairment of their immune function, hence an altered drug metabolism or drug interactions.

**Sample size determination:** we used the following formula to estimate the sample size:

$$N=\frac{\frac{r+1}{2r}(P^{-})(1-{\text{P}}^{-}){\left(2\text{β}+2\text{α}\right)}^{2}}{{\left(P1-\text{P}2\right)}^{2}}$$

r = ratio of controls to cases P1 = Proportion of individuals among controls exposed P2 = Estimate of proportion of cases exposed α = 0.05 β = 0.2 Confidence level = 95% Power = 80% Ratio of control to case = 2.

A study, from South Africa 2008, reported that 25% from ART-exposed HIV patient have drug resistant strains due to lack of adherence [17]. Hence, percentage of controls exposed = 25% Odds ratio = 4. Using above assumptions, we got number of cases with exposure as 62. Therefore number of control = 124. Because of attrition, we added 10% making our sample size 68 cases and 136 controls.

**Sampling technique:** the participants were recruited on the clinic days using simple random sampling method. We recruited 10-14 patients daily during the clinic days as they come into nursing station for body weight measurement, recruiting every other patient. The clinic runs three times in a week and we started with IDH Kano. The recruited clients were retrospectively assigned as cases or controls based on CD4 cells count test. Recruitment continued until our sample size was reached for cases and controls.

**Data collection:** interviewer-administered questionnaires were used to collect clinical data from all the consenting clients. Laboratory testing for the CD4 cells count was done using the BD FACSCount system available at the study centers.

**Data analysis:** data were entered into MS Excel, exported into Epi-Info 3.4.3 (US Centers for Disease Control and Prevention) software and analyzed to generate descriptive and inferential statistics such as frequencies, proportions for univariate analysis and Odds Ratio (OR) and Confidence Interval (OR) for bi-variate and multivariate logistic regression analysis. Results were described using OR at 95% CI. Poor adherence was the outcome variable, while the predictor variables were income, education level, sex, disclosure, partner sero-discordance and stigma.

**Ethical consideration:** a provisional ethical approval was obtained from Kano State Hospitals Management Board dated 22nd December 2014 with reference number HMB/GEN/488/VOL 1. The approval was premised on conducting the research within 3 months and sharing preliminary results with the Board within 6 month and final approval was given before the end of the study. The purpose of the study was explained to the patients and their freedom to participate, before obtaining their oral and written consent. Names of the participants were not included in the questionnaire and data was restricted to only those related to the research for the sake of confidentiality.

## Results

### Socio-demographic/univariate analysis

The majority of the study participants were females 142(70%) and 155(76%) were aged = 30 years, mean ages for cases was 36 years standard deviation (SD) ± 8.8 years and 37 years for controls (SD ± 9.8 years). There was no significant difference between the mean duration on ART between cases (4.5 years SD ± 2.2 years) and controls (4.9 years SD ± 2.2 years) Table 1.

**Table 1:** Socio-demographic characteristics of HIV patients receiving ART in Kano State (cases and controls)

Variable N=204		Cases	Controls
Mean Age		36 (SD=8.8)	37 (SD=9.8)
Sex	Male	25 (40)	37 (60)
	Female	43 (30)	99 (70)
Marital status	Married	42 (20.6)	80 (39.2)
	Single	8 (4.0)	10 (4.9)
	Separated	4 (2.0)	10 (4.9)
	Divorced	6 (2.9)	8 (3.9)
	Widows	8 (4)	28 (13.7)
	No formal education	15 (22.1)	70 (51.5)
Income	Above min wage ($50)	17 (9.4)	18 (10)
	Below min wage	43 (23.9)	102 (56.7)

### Bivariate analysis

Those who had formal education had 4.12 times higher likelihood of having poor adherence (95% CI = 2.1-8.1) as compared to those who had no formal education. Clients with monthly income above minimum wage had 2.24 times more likelihood of having poor adherence (95% CI = 1.1-4.8) than those with monthly income below minimum wage. Clients with felt stigma had three times more likelihood of poor adherence (OR = 3.0, 95% CI = 1.2-7.2) than those without felt stigma. Partner disclosure (OR = 0.3, 95% CI = 0.1-1.1), female sex (OR = 0.6, 95% CI = 0.3-1.2) and partner sero-discordance (OR = 0.6, 95% CI = 0.3-1.2) were found to be associated with good adherence, although the statistical association was not significant.

### Logistic regression analysis

Adjusting for sex, stigma and partner disclosure showed income above minimum wage, (adjusted odds ratio [aOR] = 3.03, 95% CI = 1.1-4.8) and having formal education (aOR = 2.38, 95% CI = 1.5-6.0) were statistically significant predictors of non-adherence (Table 2). The commonest reason for missing drugs among the cases was forgetfulness (58%) (Figure 1).

**Table 2:** Bivariate and Logistic Regression analyses for the predictors of adherence among HIV patients in Kano, May 2015

Variable		Cases	Controls	Odds ratio	95%CI	P-value	aOR	95% CI	P-value
Sex	Male	25 (40)	37 (60)	1.5	0.34-1.19	0.16			
	Female	43 (30)	99 (70)						
Education	Formal education	53 (77.9)	60 (48.5)	4.12	2.11-8.05	**<0.001**	3.03	1.5-6.0	**0.0017**
	No formal education	15 (22.1)	70 (51.5)						
Income	Above min wage ($50)	17 (9.4)	18 (10)	2.24	1.06-4.76	**0.03**	2.38	1.01-5.50	**0.04**
	Below min wage	43 (23.9)	102 (56.7)						
Partner disclosure	Not disclosed	6 (9.0)	4 (3.0)	3.3	0.08-1.1	0.07			
	Disclosed	61 (91.0)	129 (97.0)						
Stigma	Stigma	13 (19.1)	10 (7.4)	3.0	1.23-7.20	**0.008**			
	No stigma	55 (80.9)	126 (92.6)						
Partner sero-discordance	Sero-concordant	27 (75.0)	43 (66.2)	1.5	0.26-1.62	0.18			

## Discussion

We conducted this study to identify the predictors of non-adherence to ART among HIV patients attending secondary health care facilities in Kano State. Our study found that having formal education and income above minimum wage in our setting were determinants of non-adherence and hence poor treatment outcome. Also forgetfulness accounts for the major reason why our clients miss their drugs. Having formal education as a predictor of non-adherence was consistent with findings by Uzochukwu B *et al*., [18] from southeastern Nigeria but is inconsistent with findings of most studies in both resource-rich and resource-poor settings [8, 10, 12]. Further higher income as a determinant for non-adherence was reported by Njideka E *et al* from southeastern Nigeria. Having formal education and or high income are is associated with a feeling of high self-esteem. Such individuals detest to be associated with PLWH and may live with constant fear of disclosure and stigma.

Our study shows that fear of stigma is associated with non-adherence (OR = 3), supporting previous studies especially in resource-poor settings [10, 11]. Partner non-disclosure was also found to be consistent with non-adherence; a finding supported by Iliyasu *et al.*, from Kano, [13] Grace *et al* from Keffi Nasarawa State, north-central Nigeria [11] and Issifou *et al*., Togo Republic [19]. In the resource-rich setting, disclosure has not been identified as a determinant of good adherence. A study from South Africa showed that those who suffered discriminatory experience are more likely to be non-adherent [12]. Partner sero-concordance was found to be associated with non-adherence. It is very likely that sero-discordant couples enjoy better support from their partners than the sero-concordant.

Most studies especially in the developed countries implicate use of alcohol as one of the reasons for non-adherence. None of our study participant indicated use of alcohol as a reason for missing drugs and this is in keeping with previous studies in northern part of Nigeria [14, 15]. Evidently, use of alcohol is inconsistent with the ethno-religious values in Kano State and it is currently under ban by the state Sharia laws introduced in the year 2000.

The most commonly mentioned reason accounting for missing drugs among HIV patients in our setting was forgetfulness. This attribute is nonetheless found to be more prevalent among controls indicating that the cases possess certain characteristics that may lead to non-adherence behavior different from control. The second commonest reason for defaulting among the cases were reasons not included in our questionnaire and were recorded as others. Reasons recorded under this category included feeling of frustration, travel, fasting, being too busy, misunderstanding of dose regimen and being a victim of Boko Haram terrorism. One thing common to these clients is the fact that they all have difficulty accepting their HIV diagnosis.

Our findings also suggested that male is a risk factor for non-adherence at the significance level of 0.16. This is contrary to findings from a study conducted by Uzochukwu from southeastern Nigeria whereas Iliyasu from Kano found that sex had no significant influence. The number of female clients attending ART clinic disproportionately outweigh the number of males. This is evident from the preponderance of females to the tune of 70% among the study participants. Males in our setting hardly come to routine clinics and usually get their refill drugs from their wives under the pretext of being at work.

Others get supplied through health workers acting as touts for financial gain; hence, vital services like adherence counseling are inadvertently missed. Again males are more likely to travel, have a busy schedule or omit taking drugs in the presence of others to avoid disclosure. Since lack of formal education is a consequential finding in this study, one major limitation here is that we did not seek to recognize the literacy level of the study population based on ability to read and write the Arabic text that is common in our setting. In this regard, lack of formal education may be misconstrued to mean illiteracy. Such information may provide the basis for the choice of the literature of communication (English or Arabic). However, this study affords a basis to improve in the future having understood the prevalent education level.

## Conclusion

People with high income and higher education were found to be non-adherent. We, therefore, recommend that adherence counseling should focus on all clients regardless of their social class. Individuals identified with fear of stigma shall also receive more sessions of adherence counselling. Clients should be counselled on how to use their drugs whilst they are fasting. Use of reminders like mobile phones, treatment support partner or use of pamphlets should be adopted to mitigate forgetfulness.

### What is known about this topic

- Adherence is critical to successful ART;
- Adherence behavior can be influenced negatively by feeling of stigma, non-disclosure and forgetfulness.

### What this study adds

- People with higher level of education were found to be non-adherent;
- People with higher income were also found to be less adherent compared to those with lower income.

## Competing interests

The authors declare no competing interests.
